# Policy responses to the COVID-19 pandemic in the Manitoba grocery sector: a qualitative analysis of media, organizational communications, and key informant interviews

**DOI:** 10.1186/s12889-022-13654-3

**Published:** 2022-06-21

**Authors:** Natalie D. Riediger, Joyce J. Slater, Kelsey Mann, Bhanu Pilli, Hannah Derksen, Chantal Perchotte, Avery L. Penner

**Affiliations:** Department of Food and Human Nutritional Sciences, Faculty of Agricultural and Food Sciences, University of Manitoba, 209 Human Ecology Building, Winnipeg, MB R3T 2N2 Canada

**Keywords:** COVID-19, Pandemic, Grocery, Food security, Income support, Policy

## Abstract

**Objectives:**

The COVID-19 pandemic has impacted all aspects of the food system, including the retail grocery sector. We sought to (objective 1) document and (objective 2) analyze the policies implemented in the grocery sector during the first wave of the pandemic in Manitoba, Canada.

**Methods:**

Our qualitative policy analysis draws from organizational communications (websites and social media) (*n* = 79), news media articles (*n* = 95), and key informant interviews with individuals (*n* = 8) working within the grocery sector in urban and rural, Manitoba. Media and communications were extracted between March 9-May 8, 2020 and interviews were conducted in July–August, 2020.

**Results:**

Newly implemented policies due to the pandemic fell under four inter-related themes: *Employee health and wellbeing*, *Safety measures*, *Operational measures*, and *Community support*. Employee health and wellbeing included sub-themes of financial and social support, health recommendations and protocols, and new employee guidelines. Safety measures encompassed numerous policies pertaining to sanitation, personal protection, transmission prevention, physical distancing, and limiting access. Overall, new policies were discussed as effective in making grocery shopping as safe as possible given the situation. *Compliance and enforcement*, *employee teamwork*, and *support for employees* were key themes related to perceptions of policy success in a challenging and inequitable context. Nevertheless, *government support and communication* was needed as well to ensure safety within the grocery sector.

**Conclusions:**

The grocery sector reacted to the pandemic with the swift implementation of policies to address food supply issues, prevent transmission of the virus, support their employees as essential workers, and better serve high-risk populations.

## Introduction

Canadian provinces announced widespread social distancing and various degrees of lockdowns shortly after the World Health Organization declared COVID-19 a global pandemic on March 11, 2020 [1]. See Table 1 for a Manitoba-specific timeline of key milestones and dates. While many services and businesses were closed during this period, grocery stores were deemed an essential business, and therefore eligible to remain open to the public. With the closure of workplaces, schools (and their associated meal programs), and restaurants, Canadians relied more heavily on grocery stores than pre-pandemic, contributing to food supply disruptions [2] and an increased prevalence of food insecurity [3]. Moreover, people were engaging in stockpiling of food, toilet paper, and sanitizing materials out of fear of shortages [4–6].

**Table 1:** Key dates and milestones during the first wave of the COVID-19 pandemic in Manitoba

Date	Milestone
March 12, 2020	First case of COVID-19 reported
March 18, 2020	Suspended visitors to acute care hospitals
March 20, 2020	Province of Manitoba state of emergency declared
March 20, 2020	Daycares and preschools close for non-essential workers
March 23, 2020	Schools close for remainder of school year
April 1, 2020	All non-essential businesses close
April 2, 2020	Highest number of new cases reported during the 1^st^ wave (40 cases)
May 4, 2020	Non-essential businesses re-opened (under strict guidelines)

In addition, Canadians were experiencing growing anxiety surrounding viral transmission through aerosolization and fomites [7]. Concern for contagion was particularly pronounced among those considered high-risk for adverse outcomes, as well as front-line workers in contact with the public, such as grocery workers, who were deemed “essential” [8]. This concern fueled demand for grocery delivery/pick-up services, and fewer visits to the grocery store to limit exposures, leading to larger purchase volumes [9, 10]. Furthermore, many people were required to self-isolate for 14 days, including those returning from international travel, inter-provincial travel, and those identified as a close contact of a positive case [11, 12]. Self-isolation prevented individuals from procuring food in person, leading to further demand for grocery delivery or access through a third party (i.e. family or friend).

Unlike many essential healthcare professionals exposed to the public, those in the grocery sector are paid considerably less, at an average of $17.45/hour in Canada, as reported in 2019 [13], with a likely lower average in Manitoba due to having the third lowest provincial minimum wage [14]. This translates to an average of $2,792 for a 4-week period for a full-time employee (40 h per week) before taxes and other deductions, as compared to the $2,000 per 4-week period provided by the Canada Emergency Response Benefit (CERB) [15]; though many employees in the grocery sector do not work full-time [16]. CERB was offered beginning April 6, 2020 for Canadians who had lost employment or diminished income to ensure there were limited financial barriers for Canadians to stay-at-home [17]. The benefit was accessed by approximately 29% of Manitoba workers between March and September 2020 [18]. This further contributed to challenges with respect to retention of the grocery workforce at a time when demand and workload were increasing, which necessitated temporary wage increases [19]. An analysis of temporary wage supports is critical.

Research into the impact of COVID-19 on the grocery sector is growing (e.g. see Brandtner et al. [20], Crowell et al. [21], Li et al. [22]), including research and discussion based on a Canadian context (e.g. see Goddard [10]). However, we are not aware of any research including media sources focused on the grocery sector, as well as research focused on a Manitoba context, including rural perspectives. Mass media communicates issues of relevance to communities and are a critical element of public health communication; the public responds to public health issues based on how information is communicated by the media (e.g. see Mackay and colleagues [23]). Rural populations, while heterogeneous, have emerged as an at-risk population for COVID-19 mortality in the U.S [24]., as well as being significantly more vaccine hesitant in Canada [25]. Therefore, how rural grocery stores, workers, and communities may have experienced and/or perceived the first wave of the COVID-19 pandemic is highly relevant.

Given this context, the grocery sector implemented many operational changes in a short period of time (and prior to direction from public health) to prevent infection, while adjusting to rapid changes and challenges in food procurement and supply, and supporting their employees. It is critical to document these operational changes within the sector and conduct a policy analysis to learn what worked, and why it worked, to inform future responses in similar emergency situations. Furthermore, a policy analysis will provide an important description of the occupational health and safety of grocery employees, who are a critical occupational group in maintaining food access. This analysis may inform health and food policies globally as well as future research, as we collectively respond to, and continue to learn from, the global pandemic and its impact on the retail food sector and its employees, and ultimately public health. Therefore, we sought to (objective 1) document the policies implemented in the grocery sector during the first wave of the COVID-19 pandemic; and (objective 2) analyze the policies implemented.

## Methods

### Design

**Fig. 1:**
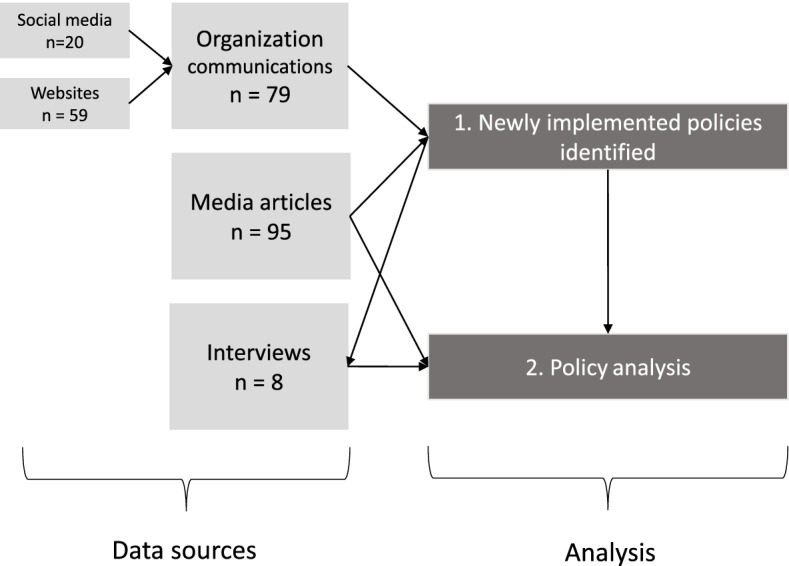
Summary of data sources and flow of analysis

Our sequential, qualitative policy analysis draws from organizational documents (i.e., website communications), Canadian news media articles, and key informant interviews with individuals working within the grocery sector (Fig. 1). Our analysis was guided by the National Collaborating Centre for Public Health Policy (NCCHP) analysis framework to document and analyze the intended effects, unintended effects, and equity of the effects, as well as issues related to implementation, including cost, feasibility, acceptability, and durability of the policies [26]. An initial analysis from organizational documents and news media to identify and extract policies implemented (objective 1) further informed the interview guide and opportunities for additional probing during key informant interviews to analyze the policies implemented, together with the news media articles (objective 2).

### Setting

Manitoba is located in central Canada and includes a population of 1.3 million, with 72% residing inside a central metropolitan area, which is lower than the national average [27]. Rural and remote areas disproportionately include First Nations populations, people working in the agricultural sector, and includes residences for recreational purposes (for example, cottage and lake areas).

### Data collection

#### Organizational communications

**Fig. 2:**
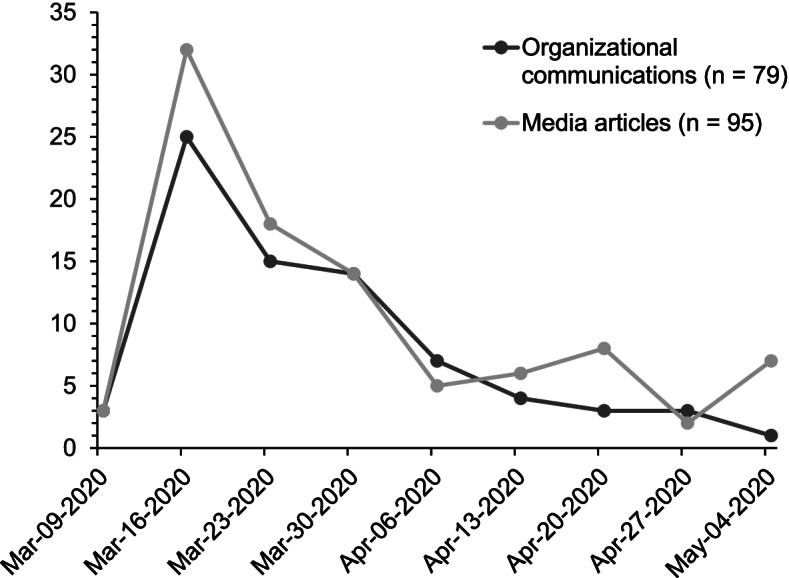
Timeline of media publications and organizational communications, presented as weekly totals

A research assistant (KM) conducted a systematic search of website communication specific to the COVID-19 pandemic from large national chain grocery stores and the parent company, as well as Manitoban independent grocery stores (Table 2). To ensure we captured communications from independent local stores in Winnipeg, we included website and social media (Facebook) communications from four Winnipeg-specific stores. Websites were visited weekly to ensure communications (i.e. posts/memos/letters) were documented as well as the date announced, where possible. Communications for all these businesses were extracted between March 9, 2020 and May 8, 2020. These dates were selected as March 9, 2020 was before any of the previously listed businesses provided communications regarding COVID-19, and there were few communications or new policies/procedures being enacted by the beginning of May leading to fewer communications (Fig. 2).

**Table 2:** Business/stores included in search for organizational communications on websites and social media

Store type	Parent company (if applicable)	Individual store name(s)
Large chain grocers	Empire	Safeway, Sobeys, IGA, and FreshCo
	Loblaws	Superstore, Shoppers Drug Mart, Zehr’s, and No Frills
	The North West Company	Northern and North Mart
	N/A	Walmart, Whole Foods Market, Vita Health, Co-Op Cost-Co, Giant Tiger, Save on Foods
Independent grocers (Winnipeg)	N/A	Dakota Family Foods, Dino’s Grocery Mart, Downtown Family Foods, Food Fare

### News media

A Research Assistant (BP) conducted a systematic search for media articles related to the grocery sector and COVID-19 from CBC News, CTV News, Global News, and Winnipeg Free Press between March 9, 2020 and May 8, 2020. These media sites were included due to their national presence as well as being publicly available, with the exception of the Winnipeg Free Press, which was included to capture local Manitoba perspectives. Each news website was searched regularly using the search terms ‘grocery stores’, ‘groceries’, and ‘grocery shopping’ to identify eligible articles published within the time frame. Articles were eligible if they addressed grocery stores, food availability, restrictions impacting the grocery sector, policies implemented in the grocery sector, and public opinions relating to the pandemic and procuring food. All articles were documented and organized according to date published (Fig. 2).

### Interviews

Three trained student RAs (HD, ALP, and CP) conducted semi-structured qualitative interviews with employees working in large national chains and independent stores, as well as within rural and urban areas in Manitoba. Eligibility criteria included any adult (≥ 18 years old) working in a grocery store in Manitoba for at least the past 6 months and ≥ 20 h per week. Participants were recruited by poster, which was circulated on social media. We also used purposive sampling to target grocery/retail store managers or assistant/associate managers by contacting eligible individuals by email and/or phone to invite to participate. Interview questions focused on participants’ work prior to the pandemic; their store, services, employees, and customers; their work experience during the pandemic thus far; challenges encountered; policies implemented; lessons learned; among other topics. Additional probing questions were integrated into the interview guide to better understand challenges or experiences in implementing *specific* policies and how their customers and employees responded. Interviews were conducted via telephone in July and August 2020 and transcribed verbatim. This data collection was approved by the University of Manitoba Joint Faculty Research Ethics Board (HS23899) and all participants provided informed consent.

### Analysis

Data, including organizational documents, media, and interviews were analyzed using the framework method described by Gale and colleagues [28]. The framework method is informed by thematic content analysis, is well-suited for large amounts of data from different sources, when an analytical framework can be pre-determined (as was the case with the NCCPH: effectiveness, equity, cost-effectiveness, etc.), and for use with multiple coders. All analysis was completed in Microsoft Excel.

First, all documents/communications (i.e., websites) were reviewed by an RA (KM) with any policies identified entered in separate rows (each policy was a discrete ‘case’). For each case, we entered the organization, a ‘store type’ dichotomized as large national grocery chain or local/provincial independent store, the date the policy was implemented if available, and a verbatim description of the policy, extracted from the document. A second RA (BP) completed a review of 10% of the documents to ensure consistency and reliability in this data extraction. Any discrepancies in coding were resolved by meeting with the first and second authors to achieve consensus. At this stage, policies (i.e., cases) were collapsed into categories whereby similar policies from different stores/chains were grouped together (second-level cases).

Second, all media articles were reviewed by a Research Assistant (BP) to identify any policies/procedural changes in grocery stores that were discussed in the text. A second Research Assistant (KM) completed a review of 10% of the media articles to compare the policies extracted to what were previously identified to ensure reliability in coding. Any discrepancies were resolved by discussing with the first and second authors to achieve consensus. Once all the policies/procedures were documented, from both documents and news media, similar policies were grouped into *policy themes* by two RAs (BP and KM) and reviewed by the two lead authors. Notably, some policies/procedures appeared in multiple policy themes.

Third, news media articles were used to analyze each *policy theme* (row) according to six pre-determined, initial codes (columns): context, intended effect, unintended effect, equity, feasibility, cost, and acceptability [26]. Specifically, we analyzed the data to answer research questions, such as, was this policy perceived as effective, (in)equitable (if so, for whom?), cost-effective, etc.? Was there something about the context than influenced perceived effectiveness, cost, etc.? Text was extracted from media articles within this framework, followed by open coding of this extracted text in subsequent rounds of analysis by the lead author (NR). In this way, analysis was both deductive and inductive to allow space for unexpected or contextual phenomena that were relevant to our analysis of the policies, but ultimately guided by the NCCHP policy analysis framework. These codes were then applied to interview transcripts by another author (KM), with new codes added iteratively, and also applied to the media analysis through ongoing communication between the two coders to refine the code list and multiple iterations of coding. Finally, codes were grouped into second- and third-level codes, merged into categories, and themes were constructed using a mind-map through discussion and consensus amongst the authors [29]. Using a mind map allowed for connections to be presented amongst the themes to illustrate how themes related to each other.

## Results

### Objective 1: Policies implemented

We reviewed a total of 79 website/social media communications and 95 media articles related to grocery stores and COVID-19 to identify the policies implemented (summarized in Table 3). The majority of the communications and media articles were published early in the first wave, and tapered off into mid-April (Fig. 2). The policy themes are *employee wellness and supports*, *safety measures*, *operational measures*, and *community support*. *Employee health and wellbeing* included sub-themes of financial and social support, health recommendations and protocols, and new employee guidelines. *Safety measures* encompassed numerous policies pertaining to sanitation, personal protection, transmission prevention, physical distancing, and limiting access. New *operational measures* were implemented due to increased demand, particularly for delivery/online orders. Finally, *community support* was enacted through policies to enable improved access for high-risk/priority groups and enhance charitable food contributions.

**Table 3:** Description of thematic analysis of grocery policies/procedures

Policy Theme	Sub-themes	Categories/examples
Employee wellness and supports	Financial supports	Wage supports for employees (e.g. Increased wages, bonuses, financial assistance while self-isolating) Benefits for sick time Benefits for part-time employees
	Social supports	Medical supports (e.g. Access to online healthcare) Social supports (e.g. Associate discount enhanced)
	Protocol	Stay home if sick Protocols for positive employee COVID-19 test Employee health and wellness checks (e.g. temperature checks) Task force to create protocols and employee responsibilities Employee safety training and compliance
	Employee guidelines	Travel guidelines for employees Office employees to work remotely
Safety measures	Sanitization	Increase surface sanitization Increase employee sanitization Implement customer sanitization
	Personal protection	Wear masks (employees and customers) Optional PPE for employees Installation of protective shields at checkout
	Preventing spread	Policies regarding use of plastic and reusable bags Cash-free transaction recommendations Limit person-to-person contact (e.g. Customer bagging, contactless delivery)
	Limited access	Limit customer/store capacity Limit entry/access points and public facilities Limit in-store customer idling
	Physical distancing	Visual measures for physical distancing (e.g. Floor markers) Spatial measures for physical distancing (e.g. Opening every other check-out counter) Verbal reminders for employees and customers Employee enforcement of customer physical distancing
Operational measures	Increased online/delivery capacity	Increase access to grocery delivery service Increase capacity for online grocery ordering Add resources for grocery delivery service Increase access to grocery pick up service
	Coping with increased demand	Limit number of online orders Flexibility in services offered to meet demand Limits on number of products purchased Hiring of temporary staff
	Operational changes	Product pricing (e.g. Freeze product pricing) Changes to return policy (e.g. No returns) Limit store hours to increase time for cleaning and restocking Change supply and distribution processes
Community support	Priority access	Prioritization of grocery delivery for quarantined and high-risk customers Financial incentives for high-risk customers (e.g. Senior or employee discount) Priority online access for healthcare workers Reserve shopping hours for high-risk customers (e.g. Elderly or with pre-existing conditions)
	Collaboration and outreach	Documentation and public disclosure for positive employee COVID-19 test Cease community outreach events Donations to charitable sector and launch charitable food campaign

Most policies appeared to be universally applied across Canada, with a few exceptions. The continued use of reusable bags in stores was not universally allowed initially and in British Columbia, for example, were banned. At this point in the pandemic, masks were not mandatory for customers or employees, and as such are not considered in our analysis. Also, at least one grocery chain piloted temperature checks for employees and customers, but it was unclear how this policy was received or how long the practice continued, but was critiqued in the media.

### Objective 2: Policy analysis

**Fig. 3:**
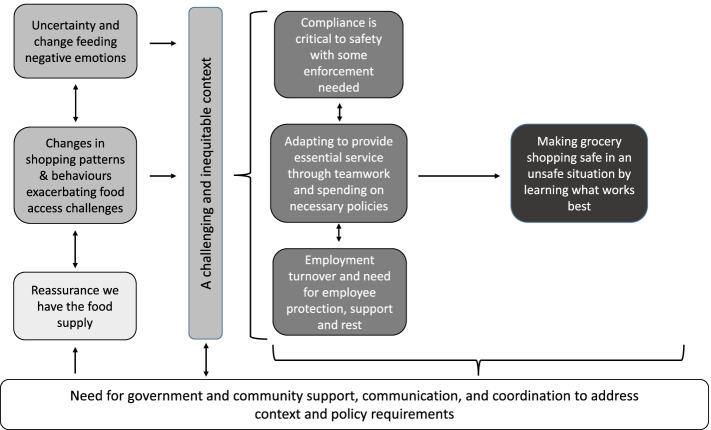
Mind map of policy analysis from media and key informant interviews

We conducted eight interviews with key informants, including four who worked in an urban setting and four in rural settings in Manitoba. Interviews ranged in length between 19 and 70 min (average 49 min). Participants worked in various roles including management, cashier, bakery department (with role shifting during the pandemic), wholesale, delivery, and stocking shelves in multiple stores. Our policy analysis is summarized as a mind-map in Fig. 3. Relevant quotes are presented with either participant number when from an interview, or the date published when from news media.

#### A challenging and inequitable context

Participants and media described a very challenging context for the grocery sector during the first wave. The COVID-19 pandemic generated fear and anxiety specific to the virus and its transmission, and sadness among customers related to job losses and school closures that fueled stress among customers and employees. The societal uncertainty regarding COVID-19, constant changing of government policies specific to COVID-19, and the impacts on grocery employees, such as rapid turnover, further added to the challenges facing the grocery sector.

When COVID first started, I'd say there was a handful of people that couldn't deal with the stress because when you see all the customers stressed out, it puts stress on you and then you feel- a lot of the people felt like they were putting their life on the line by coming to work. **–** Participant 4

COVID-19 was not the only source of fear and anxiety, there was also fear regarding food supply, particularly among customers, further adding to the stressful climate and influencing shopping patterns. Grocery shopping patterns changed rapidly through high demand fueled by school/workplace/restaurant closures, stockpiling, less frequent shopping excursions contributing to more purchases, and third-party purchasing for family/friends self-isolating or who were more inclined to stay home.

For the most part, people would come in and do small grocery shops during the week as they need it. Well now the majority of people that come in are doing a larger grocery shop every other week. – Participant 1

Relatedly, more Canadians were shopping for groceries online for delivery and pick-up. Together, these changing patterns contributed a high demand for groceries, and through different systems (in person vs. delivery/pick-up), in a very short period of time. The increased demand for specific types of foods (e.g., yeast, flour, meat products, canned pop) resulted in challenges in food access to specific products and shortage in the number of these products.

There will be shortages in every grocery store in different categories on a daily basis because demand is outstripping any way you could possibly amp up your supply. – March 17, 2020

Policies were implemented quickly to prevent transmission of the virus, which led to confusion and at times, contradicting policies. This fueled the uncertainty and fed into negative emotions of both customers and employees.

Everything is different...everything has changed, right from supply to servicing our membership. Cleaning has changed, maintenance has changed, day to day functionality has changed. – March 23, 2020

This challenging context also created inequities for both certain populations (i.e. elderly or immuno-compromised customers, and those self-isolating) and types of grocery stores (e.g. smaller stores and rural stores). Rural populations faced unique challenges, both in terms of perceived risk of COVID-19 transmission and disruptions to the food supply. We heard that rural stores recognized new customers shopping at their stores early in the pandemic due to lower perceived risk of exposures by those customers. Furthermore, workers in rural areas felt more at risk of exposure due to serving transient populations such as truckers or cottagers, as well as communities who had to travel further to procure food were also described as being more at risk of exposure.

Many northern communities don't have a grocery store. In the Cree Nation for example, Oujé- Bougoumou and Waswanipi, don't have a local community grocery store and residents must travel to larger, non-Indigenous towns like Chibougamau more than an hour away. – March 25, 2020

Some changes to customer shopping patterns (i.e. stockpiling) were short-term as the grocery sector and the public adapted to the situation. Notably, lower income populations were noted in media as being unable to stockpile. Other changes to shopping patterns suggested permanency, such as increased online shopping and delivery. The extent to which some policies and procedures were thought to be long-term, garnered greater investment of resources.

While it's "difficult to anticipate precisely how our business will change in any emerging new normal," executive chairman Galen Weston said the company believes" that online grocery shopping will retain a significant proportion of the current sales penetration.” – April 29, 2020

### Making grocery shopping safe by learning what works best

Amidst this formidable context, there was an understanding that given the essential and public nature of grocery shopping, the workplace was inherently risky, but that employers were doing everything they can to try to keep employees safe in the workplace.

Grocers are balancing the need to stay open and keep Canadians supplied with essential goods with concerns about becoming risky gathering places where the virus could spread. – March 20, 2020

In the end, media and participants both largely described grocery shopping, and working in a grocery store, as safe as it could be given the risky situation. This perception of safety was attributed to learning what policies worked best and transparency. This included learning what policies worked for grocery stores in other countries, other stores within a chain, other employees within the same store, and trying different strategies and policies to determine the best approach.

We learned that we are…. able to adjust our policies so that we can umm…our policies and the way we do things so that we can meet the needs not only of our customers but also of our staff. Umm when push comes to shove, we can make it work. – Participant 4

Large grocery chains had a policy of reporting positive COVID-19 cases in specific stores on their websites, and this was presented or viewed positively, as well as closures for deep-cleaning following positive cases. There were some caveats or factors influencing perceived safety however. These sub-themes included i) *compliance as critical with some enforcement*; ii) *adapting through teamwork and policy spending*; and iii) *employee turnover and the need for support.*

### Compliance is critical with some enforcement

For government and grocery policies to be effective in preventing spread of COVID-19, compliance of employees and customers was perceived as critical, as well as enforcement in situations when compliance did not occur.

Union leaders representing workers in the grocery store and essential service sectors are pleading with the public to respect policies meant to slow the spread of COVID-19. – March 27, 2020

Enforcement of policies was associated with its own set of challenges, particularly among customers. Media and participants described negative reactions from some customers in enforcing policies.

CBC News has also spoken with a number of grocery store employees who say they have been verbally abused by angry, frustrated and impatient customers. They say they have been yelled at, cursed at, and accused of overreacting as they try to enforce physical distancing measures put in place by their employers. – April 2, 2020

This resistance further added to the stress and anxiety for many employees. Largely though, non-compliance was described as the exception as most customers were perceived as following the rules, and some policies as working with an honour system.

Uhh well we have priority access, first shopping hour of the day for elderly or people needing extra time or immune compromised people. We still have that, but it's not enforced in any way. We don't have any special security that uh accuses somebody of not having an ailment that they can't come in at that time, we just let whoever come in so. – Participant 4

Employee compliance with policies was discussed more positively, with any non-compliance attributed to getting used to the “new routine”.

He tries to wipe down the handheld debit machine or any other surface when he isn't punching the cash register. "It's a learning curve right now, remembering all the tasks we have to do." – April 4, 2020

### Adapting through teamwork and policy spending

The grocery sector adapted to provide its essential service through teamwork and contributing financial resources towards necessary policies. Participants and media articles were effusive in their praise for their co-workers, employees, and to a lesser extent their employers. This teamwork included rearranging duties and work hours, being open on holidays, going above and beyond regular duties, and kindness.

What I think I've learnt the most is how people [sighs] even when they just work with - work together you, you know, you have it it can become like a family but this - this to me has has gone even a step above that. There's nothing that nobody wouldn't do for each other. Um I think that's that's one of the things that I really have learned is how kind people really are when it comes down to possibly catching something that can kill you, do you know what I mean? – Participant 6

The teamwork and contribution of an essential service during a public health emergency contributed to a great deal of satisfaction among employees. This satisfaction and appreciation helped buoy employees during stressful times and one participant described how they hoped the appreciation they felt during the first wave would last.

And often grocery store workers, um sanitation workers, a lot of the people that have now been deemed essential…They seem to have been those ‘oh well, if you don’t have an education go work at a grocery store’ or sort of ‘lesser than’ jobs by some people. And now, all of a sudden, everybody was like ‘oh my God, thank you, thank you, thank you, thank you’. I really hope that it doesn’t go back to people thinking that they are ‘lesser-than’ jobs. They are very much needed jobs…we need those people in all sectors, all jobs are important. – Participant 3

In addition to teamwork, it was clear the grocery sector invested a significant amount of resources on policies (such as plexiglass at cash registers) to keep everyone safe and wage supports to employees. Though, this was nearly always mentioned in the context of a significant amount of revenue being generated by the grocery sector during the first wave.

Loblaw is spending about $90 million every four weeks on efforts to keep its stores safe for customers and employees, on temporary wage increases and other measures, Myers said. "It's incredibly hard for us to predict the future after all this," he said when asked how much of those cleaning, extra staffing hours and other costs Loblaw expects to become embedded permanently in its operations. – April 29, 2020

### Employee turnover and the need for support

Wage supports for employees were one of many human resource policies implemented in the grocery sector as turnover and subsequent recruitment/turnover was high. Compliance, enforcement, and the teamwork required to make grocery shopping as safe as possible hinged on employees, and supports to recruit and retain them. Many stores lost employees during initial stages of lockdown due to fear, CERB, and lack of childcare, and greater absenteeism due to self-isolation. This led to “skeleton crews” amidst higher demands.

Yes, ‘cause we had some people that went off, like as soon as they offered CERB they were gone. And umm so then I would have to step in because- or they'd be phoning in the morning saying "I have a little bit of a cough and I'm not coming in" and then... Then you just have to take over like you'd just have to run the store. It doesn't matter what job you do, right. – Participant 6

In order to support employees during the first wave, some stores reduced hours to allow employees to rest and/or have dedicated time to restock shelves, for example. As well, employees needed protection and personal protective equipment that was in short supply. This led union leaders to request that grocery workers be designated “essential”, allowing them to access limited supplies and services.

He wrote a letter to Premier Brian Pallister on Thursday asking him to declare grocery stores, food production and food distribution an essential service during the COVID-19 pandemic. Workers in these industries should be given protection similar to that of health-care workers when it comes to gear such as gloves, masks and cleaning supplies, UFCW's letter said. Those workers also need to be provided options for accessible childcare, he said. – March 19, 2020

The most discussed employee policy in the media, and interestingly, to a lesser extent in the interviews, was the wage support programs. Importantly, while wage support programs were eventually discontinued, this did not occur during our time frame for collecting media articles, but during the time interviews were conducted. The wage supports were largely lauded in the media and some participants described them very positively. Participants differed in their perspectives regarding the ending of wage supports. One participant was fairly neutral in describing the wage increase and stoppage, and another commented that they work “just to get out of the house”, not for money. Others described co-workers as being upset at the abrupt ending. Participant 4 specifically described being offended, particularly regarding perceived inequities in the bonuses.

... they made a mistake calling us heroes cause heroes don't stop being heroes. If you're gonna call us that then- and anyways and then they made- the very, very first thing when they said that we're gonna get that bonus in pay, they actually released what they're giving their supervisors and people that are, you know running the departments, and it was exponentially more that they were giving them. – Participant 4

### The need for government and community support

The challenging context required government and community support, and communication. Given panic buying, changes in shopping patterns, and disruptions to the food supply, it was critical that government and food industry reassure Canadians that we had a sufficient food supply and caution against panic buying. This served the purpose of diminishing anxiety surrounding the food supply, minimizing panic buying, and in turn addressing some safety concerns for employees and customers, as well as downstream impacts on the charitable food sector and food insecure populations.

The Alberta government is reassuring people there isn’t a food shortage in the province and that it’s keeping an eye on demands in rural areas, Indigenous communities and food banks. – March 26, 2020

Independent stores lacked the resources that larger chain retailers had to learn about what works best and implement policies, and were more reliant on government to provide direction on mandates for public health precautions. Notably, independent stores are disproportionately located in rural areas. Grocers largely operated without direction from government initially to deal with the pandemic.

Neault said some stores have taken it upon themselves to be as safe as possible for workers and shoppers alike, but some stores have not. "We just believe that there should be one set of regulations that fits everyone so that all workers and the community can feel safe," he said. – April 21, 2020

Also, other public health measures exacerbated the inequitable context for rural and independent grocery stores, who perceived greater risk of exposure due to servicing more transient populations (e.g. cottagers, Indigenous populations, returning travelers, and truckers). On the other hand, independent grocers discussed being able to pivot more quickly to delivery methods, given their smaller operations.

The grocery sector also relied heavily on the community and various community groups/organizations to facilitate grocery delivery when services were overwhelmed or shop for others who were not able to shop in person (i.e. elderly, immunocompromised, and those self-isolating). Finally, concern was also raised about individuals who were not following public health orders to self-isolate (e.g. due to symptoms or travel) and instead frequenting grocery stores, again seeming to largely effect rural stores with respect to returning travelers. Much like many grocery-specific policies, employees and citizens were left to plead with the public through media to encourage compliance with government policies.

There are growing concerns in a small Manitoba community near the American border. Pat Schmitke, who owns Morris Bigway Foods, said Canadians coming home from the U.S. are stopping and shopping when they should be heading straight home to self-isolate for two weeks. “It seems to be that this is the first stop on their way back, and they’re stopping to pick up groceries and supplies, and they’re not heeding the warnings to stay out of the stores and self-isolate,” said Schmitke. – March 26, 2020

## Discussion

The grocery sector reacted to the pandemic with the swift implementation of policies to address food supply issues, prevent transmission of the virus, support their employees as essential workers, and better serve high-risk populations. Critical to reducing risk of spread in stores initially, was addressing perceptions regarding food shortages to lessen the number of people congregating in stores to access food and panic buying, as reported by others about Canada [2], as well as globally [30]. The government, the grocery sector, and the wider food industry, were instrumental in conveying this message via media. However, it is important to note that food supply was pressured by consumer factors other than panic buying as well, as we reported in our introduction here and as well as others globally [31–37]. Regardless, we should not take for granted the importance of having an adequate food supply in minimizing transmission of COVID-19. In contrast, for lower and middle-income countries, where access to available food is more constrained, lockdown policies were thought to aggravate food insecurity to an unmanageable extent [38].

Policies directed at income-supports is an important issue that requires discussion and further analysis to inform responses to any future emergencies. First, we provide evidence of perceptions of employees leaving the grocery sector due to the CERB. While temporary wage supports for the grocery sector were necessary to recruit and retain human resources, and were advocated for strongly by unions, the extent to which wage supports have directly contributed to now rising food costs [39] is unclear. Notably, the CERB was lauded by some groups as an important indicator of the need for, and feasibility of, a guaranteed minimum income (e.g. UBI Works [40]). The CERB, as well as guaranteed minimum income, are a floor, and do not necessarily address income *inequalities*; therefore, these policies’ impact on the food system requires examination given the low wages in the sector, and Canadian families’ growing food budgets [39]. On the other hand, potential increased spending on food, stimulated by income supports (such as CERB and ‘hero pay’), may mitigate any increased food costs stimulated by higher grocery sector wages. Relevantly, increases in minimum wage are associated with purchasing more calories [41]. It must also be noted, given the increase in food insecurity, that the flexibility of Canadians to spend their CERB supplement according to their needs (food and otherwise) is likely a more feasible, ethical, efficient, and effective approach to addressing unemployment-related food insecurity and pandemic-related supply shortages than programs providing vouchers for specific foods (e.g. the American WIC program [42]).

Customer compliance and enforcement of policies was also perceived as critical to safety and prevention of viral transmission for employees and the public, which has also been reported elsewhere [22, 36, 43]. Growing evidence supports the extent to which consumers changed their shopping behaviours [44]. Importantly, compliance and enforcement of mask mandates in grocery stores in select American counties is also associated with fewer COVID-19 cases among workers [21]. Similar to what was reported by Eriksson & Stenius [36] in Finland, we found that media and participants described customers as largely compliant, with individuals engaging in “careless” behavior as more of the exception. This also aligns with Canadian data suggesting that among various activities and locations, people are most comfortable returning to, or continuing to, shop in a grocery store [45].

However, the policies and associated behavior changes were not without costs. As reported here, grocery employees experienced significant stress. In other research, the impact of the uncertainty of the situation and fear of the virus among workers were associated with depression and anxiety [46]. Importantly though, participants in the present study discussed relationships among co-workers and the appreciation workers felt from the public contributed to more positive emotions in coping with stress, similar to that reported in Spain [47]. On the consumer side, Brandtner and colleagues [20] reported lower consumer satisfaction with grocery shopping in Austria, particularly with stores not having sufficient product availability.

A key contribution of our study is the analysis of differences in rural and urban grocery stores. Similar to others [48], we also report some consumers opting to shop at smaller grocery stores, which are disproportionately located in rural areas, sometimes located near larger urban centres. This is also reflected in research demonstrating that customer satisfaction dropped less in response to the pandemic among smaller/medium size stores compared to larger retailers [20]. In both media and interviews, we did identify some urban–rural tensions with regard to customers travelling larger distances to shop for groceries and perceived increased risk of exposure for both customers and grocery workers. This perception of increased risk may be informed by underlying and ongoing urban–rural discourses, as discussed by Weeden and colleagues [49] in the context of COVID-19.

There are a number of strengths to our study including the use of three different data sources, which were used to triangulate our findings. The inclusion of organizational documents from national grocery chains as well as independent stores, and key informant interviews in rural and urban areas within Manitoba enhance the transferability of the findings within Canada. However, the transferability of the findings outside of Canada is likely limited due to different cultural contexts, food systems, social policy (e.g. CERB), and labour unions (see Crowell and colleagues [21] for evidence regarding associations between unions and COVID-19 cases among grocery workers). In this regard, analysis of union communications may have uncovered additional information regarding COVID-19-related policies. Ongoing debriefing amongst all authors to discuss areas of divergence as well as the use of the initial policy documentation to inform key informant interviews enhance the reliability of the analysis process. However, we are limited by the inclusion of only eight participants at one point in time, who remained working during the pandemic. Perspectives of employees who left the grocery sector would provide additional, likely different perspectives, particularly given evidence that job insecurity is associated with adverse effects on wellbeing [50, 51]. This should be an area of future research.

In conclusion, the grocery sector in Manitoba implemented a number of policies and procedures to address safety and food supply, many of which will likely outlast the pandemic. Overall the retail grocery sector was perceived by participants in the present study, and presented by the media, as being as successful in making grocery shopping as safe as it could be given the pandemic conditions. Key to safety was an adequate food supply and public compliance and enforcement of safety policies, and supports for employees. We recommend earlier enforcement of product purchase limits, which would likely contribute to lesser panic buying and fewer, frequent grocery store visits to purchase products (and hence, limiting viral exposure and associated anxiety). However, our research also raises further questions, such as how wage supports will (dis)continue through subsequent waves and their impact on recruitment, retention, and employee safety. This would influence any recommendations regarding if, or how, this policy could be implemented in the future.

## Data Availability

Dr. Natalie Riediger (email: Natalie.riediger@umanitoba.ca) can make the data available upon reasonable request.

## Abbreviations

CERB: Canada Emergency Response Benefit
NCCHP: National Collaborating Centre for Public Health Policy
RA: Research Assistant
